# Hydrogel on a Smart Nanomaterial Interface to Carry Therapeutics for Digitalized Glioma Treatment

**DOI:** 10.3390/gels8100664

**Published:** 2022-10-17

**Authors:** Xinyi Zhao, Bilal Javed, Furong Tian, Kangze Liu

**Affiliations:** 1 School of Food Science and Environmental Health, Technological University Dublin, D07 EWV4 Dublin, Ireland; 2 School of Chemistry, Chemical Engineering, and Biotechnology, Nanyang Technological University, Singapore 639798, Singapore

**Keywords:** glioma, hydrogel, gene therapy, biosensor, digitalized, precision medicine

## Abstract

Glioma is considered the primary brain tumor to cause brain illnesses, and it is difficult to treat and shows resistance to various routine therapeutics. The most common treatments to cure glioma are the surgical removal of tumors followed by adjuvant chemotherapy and radiation therapy. The latest biocompatible interfaces have been incorporated into therapeutic modalities such as the targeted delivery of drugs using hydrogels to treat and manage brain glioma. This review illustrates the applications of the multimodal hydrogel as the carrier of therapeutics, gene therapy, therapeutic tactics, and glioma devices. The scientific articles were retrieved from 2019 to 2022 on Google Scholar and the Scopus database and screened to determine whether they were suitable for review. The 20 articles that fit the study are summarized in this review. These studies indicated that the sizes of the hydrogel range from 28 nm to 500 nm. There are 16 out of 20 articles that also explain the post-surgical application of hydrogels, and 13 out of 20 articles are employed in 3D culture and other structural manifestations of hydrogels. The pros of the hydrogel include the quick formulation for a sufficient filling of irregular damage sites, solubilizing hydrophobic drugs, continuously slowing drug release, provision of a 3D cell growth environment, improving efficacy, targetability of soluble biomolecules, increasing patient compliance, and decreased side effects. The cons of the hydrogel include difficult real-time monitoring, genetic manipulations, the cumbersome synchronized release of components, and lack of safety data. The prospects of the hydrogel may include the development of electronic hydrogel sensors that can be used to enhance guidance for the precise targeting patterns using patient-specific pathological idiosyncrasies. This technology has the potential to revolutionize the precision medicine approaches that would aid in the early detection and management of solid brain tumors.

## 1. Introduction

Glioblastoma multiforme is categorized as a fast-growing grade four brain glioma. It develops in star-shaped glial or non-neuron cells (astrocytes and oligodendrocytes) that do not produce electrical impulses. Glial cells play a significant supportive role to the neuron cells in the brain and perform important physiological functions. Glioblastoma multiforme (GBM) accounts for 60% of brain tumors, and the median reported survival of GBM patients after its first diagnosis is, unfortunately, only about 16 months due to the unstoppable proliferation of glioma cells, poor diagnosis, disease prognosis, and a high-grade metastasis [1]. Glioma patients are treated with surgery to remove the tumor or the tumor-carrying site, followed by chemotherapy and radiation therapy to slow or stop the tumor growth [2,3]. Various scientific studies have been carried out to discover new therapies and approaches to regulate tumor progression. These practices involve new ways to treat patients with glioma precisely due to drug resistance and recurrence of glioma tumors [4].

Recently, nano-assisted therapies such as nano-immunotherapy, nano-stem cell therapy, and nano-gene therapy have been investigated for brain cancer treatment [5,6,7]. The safety of GBM treatments has been considered as a priority. Hydrogel is a network of three-dimensional crosslinked polymers that can absorb and retain water. The biodegradable polymeric gels contain encapsulated therapeutics that can be deployed locally in the resection cavity. They have diverse physical and chemical properties which make them a suitable candidate for multiple biomedical applications. Hydrogels have emerged for efficacious intracavity drug delivery in preclinical brain tumor models to circumvent some of these limitations [5].

Hydrogels have diverse physical and chemical properties which make them a suitable candidate for multiple biomedical applications. Hydrogel is a tissue-like material that provides the system foundation to build up the next-generation flexible biosensors [8]. Hydrogels are built up with purely synthetic components or cellular–synthetic hybrid components. Tissue-like hydrogels are designed to mimic the naturally occurring biological components from the morphological and structural level, which enables them to acquire biomimetic mechanical, chemical, physical, and electrical properties [8]. The sophisticated and highly integrated tissue-like materials and systems hold great potential to achieve bio-functions and can be applied to a wide variety of body parts with different tissue types [9]. The hydrogels are considered the most promising carriers for the targeted delivery of drugs to cancerous sites and have the potential to act as an integrated biosensor interface with excellent sensitivity, accuracy, high conformability, and extended durability [10].

Hydrogels have been made up of covalently bonded subunits that define their properties. Additionally, the functional groups of the monomers provide a convenient location for loading therapeutics [10]. Hydrogels, as nanocarriers, have been designed to carry a stimulating drug delivery system [11]. Research has shown that hydrogel has the advantage to act as a carrier in chemotherapy medicine to deliver drugs using a wide range of small molecules, drugs, gene therapy, or immunotherapy using the same nanomaterial platform with minor alterations [12]. Hydrogel as a smart biomaterial has been employed to carry lipid carriers, polymer nanoparticles (NPs), metal nanoparticles (MNPs), biobased NPs, and injectable or implantable 3D scaffolds [2,12]. The scaffold/polymer layers are loaded with different therapeutic modalities used against glioma cells. Nanomaterials also allow the continuous release of many medicinal compounds in response to external stimuli such as acidic, mechanical, electrical, magnetic, light, and thermal pH [13]. NPs have been embedded in a heat-sensitive hydrogel that has the capabilities to rapidly increase temperature and help to improve the site-specific retention of NPs in tumors [2]. Low-density lipoprotein receptor (LDLR), EGFR receptors, mesenchymal–epithelial transition factor (MET), transferrin, and HER2/EGFR-tagged/decorated NPs have been invested at the site of glioma injury [14]. The nanocarriers have been coated with peptides or antibodies specific to brain endothelial cells (BEC) [11]. The hydrogel has a cell-mimicking ability to avoid the interaction of NPs with immune cells in the reticuloendothelial system (RES) resulting in phagocytosis [2]. The scaffold around the NPs can cause a slow release to provide a sustained and regulated release of cargo at the tumor site in response to different stimuli.

## 2. Result and Discussion

### 2.1. Analysis of Hydrogel as a Drug Carrier for the Treatment of Glioma

Twenty papers were identified and reviewed on Google Scholar and Scopus related to hydrogel as the drug carrier due to having the treatment properties of glioma cancer. The articles retrieved were published from 2020 to 2022. The 20 articles were evaluated or described using the investigative technique for the parameters of post-surgical or not, cell culture type (3D/2D), with/without glioma stem cell seeding, drugs delivered, particle size (nm), sensor type, pros, and cons, which are all illustrated in Table 1.

### 2.2. Hydrogel Application for the Blood–Brain Barrier (BBB)

**Figure 1:**
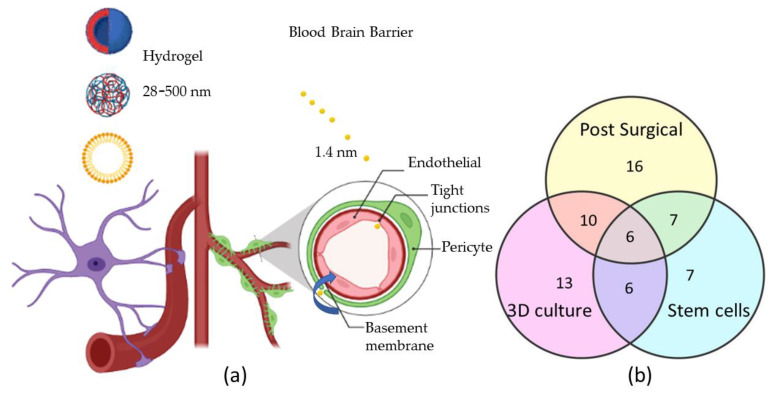
Hydrogel application for the Blood–Brain Barrier. (**a**) Illustration of small particle size and drugs under 1.4 nm can cross tight junctions and endocytosis to pass the blood–brain barrier (**b**) Article numbers of distributions in the post-surgical application, 3D cultures, and stem cells.

**Figure 2:**
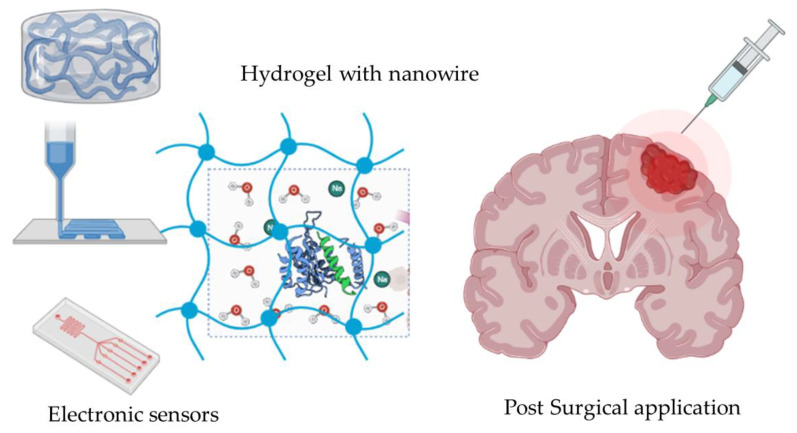
Scheme of hydrogel with nanowire for the electronic sensor.

There are many types of hydrogel, such as poly(ɛ-caprolactone)-poly(ethylene glycol) (PCL-PEG), hyaluronic acid, poly(ɛ-caprolactone-co-lactide)-b-poly(ethylene glycol)-b-poly(ɛ-caprolactone-co-lactide) (PCLA-PEG-PCLA), and poly(vinyl alcohol) (PVA) [15,16,17,18,19,20,24,32,33]. Natural products such as gelatin, triglycerol monostearate, pig diesel, and collagen have been employed as hydrogel scaffolds [25,27,29,31]. The most significant question in the treatment of glioma is what happens when nanocarriers (carrying the drugs) succeed in gaining access to the central nervous system via the BBB. The Blood–Brain Barrier (BBB) represents the structural differences that exist between the endothelia of the brain capillaries and endothelia in other capillaries, such as tight junctions between adjacent endothelial cells. The drug carrier’s size is essential for drug delivery design for glioma cancer. Semmler-Behnke et al. have reported the uptake of 1.4 nm of gold NPs in secondary target organs such as the brain following intra-tracheal or intravenous application [35]. A nanoparticle size bigger than 1.4 nm cannot pass the BBB via tight junctions (Figure 1a). There is a rare chance for the nanoparticle to pass the BBB via endocytosis and exocytosis [35]. Figure 1a has been designed to explain the anatomic structure of the Blood–Brain Barrier (BBB), and the number of articles with data included in this review on hydrogel particle size, post-surgery, cell culture, and glioma stem cell from Table 1 is incorporated in Figure 2b. The number of articles related to post-surgical application, 3D cultures, and stem cell application is against a total of 20 articles.

The particle sizes have been summarized in column 6 in Table 1. The size range of the hydrogel reported in the scientific studies was between 28 nm and 500 nm (Table 1). The designed hydrogel as a drug carrier is to maintain the drug release and does not travel through the BBB. Only the particle size of less than 1.4 nm can pass the BBB (see the blue arrow in Figure 1a). There are 16 out of 20 articles on hydrogel as a post-surgical application (Figure 2b). It is concluded that hydrogel is selected for drug release for glioma cancer treatment through local delivery by post-surgery. Local delivery of chemotherapeutic drugs (to bypass the blood-brain barrier) via hydrogels in the resection cavity is recommended [36]. The drugs can be released within the endothelium cells and undergo further transportation into the brain by diffusion or transcytosis [37]. In total, 13 out of 20 articles employ 3D culture (Figure 2b). Once administered, the anti-cancer agent drugs cross BBB. Once they cross the BBB, the specific drugs, such as carmustine, histamine, paclitaxel, temozolomide, gemcitabine, luminol, doxorubicin, thymidine kinase, and irinotecan, become attached to the specific surface markers expressed on the 3D tumor models.

### 2.3. Stem Cell Application

Hydrogel as the therapeutics carrier can enhance the drug’s penetration and retention at the tumor site. The high-water content made hydrogels suitable structures for loading stem cells [15]. There are 7 out of 20 articles employing hydrogel with stem cells for glioma cancer treatment. Of these, 6 out of the 7 articles employ stem cells in 3D model culture to treat glioma cancer (Figure 2b). Researchers have found that glioma stem cells (GSCs) and glioma cells participate in the angiogenesis of healthy glioma cells by directly transdifferentiating into endothelial cells or secreting vascular endothelial growth factor (VEGF) [8,9]. Hence, there is an urgent need to establish an in vitro or in vivo glioma model to investigate the role of glioma cells and GSCs in healthy glioma angiogenesis. The encapsulated human mesenchymal stem cells (hMSCs) could produce brain-derived neurotrophic factor (BDNF) (BDNF–hMSCs), enhancing neuronic functional recovery by reducing the neuronal death rate in the hippocampus [18]. It is generally believed that angiogenesis is beneficial to tumor progression and migration, and anti-angiogenesis is a common strategy for tumor therapy. Invasive glioma cells are resistant to clinical standard-of-care chemotherapy, along with radiation [15]. The emerging molecular target implicated in invasive glioma biology is the TNF receptor superfamily member named fibroblast growth factor-inducible 14 (Fn14) [5,38]. Additional, mural-like tumor cells differentiate from GSCs. The mural-like tumor cells significantly contribute to the microvasculature of glioblastoma. Flk-1 (human counterpart, KDR) tyrosine kinase is one of the two receptors for Vascular Endothelial Growth Factor (VEGF). Fn14 and FIK-1 could complement current anti-angiogenic treatment [39]. Therefore, stem cell transplantation by hydrogels could be a potential strategy for the clinical treatment of brain disorders.

### 2.4. Hydrogel Carrier for Gene Therapy

Hydrogel carriers as smart materials platforms have been developed for effective lung cancer treatments. Synthetic cancer-sensing circuits have been designed to recognize cancer cells based on intracellular gene expression profiles [8,11]. PI3K pathway inhibitors loaded with liposomes, cationic liposomes containing the TP53 gene plasmid, siRNAs against EGFR and PDGFRA, siRNAPVT1, siRNAp53, siRNASTAT3, and PDL1 have been investigated for glioma therapy [40,41,42]. Multi-colored RNA circuits (generating tumor suppressor microRNAs targeting key glioma driver genes) have been employed in nanohydrogels to produce the circuit’s logical synthetic genetics. This allows for the fine-tuning of both the hardware platform and the genetic circuitry, as well as a patient-by-patient study of the platform’s effectiveness in xenograft models derived from glioma patients. Researchers can easily detect and quantify tumor heterogeneity by evaluating treatment outcomes in each cell type that makes up the tumor microenvironment [43]. The multi-colored MicroRNA’s (miRNA’s) circuits can be specifically expressed in each cell type of the tumor microenvironment (cancer cells, normal cells, immune cells, tumor-associated fibroblasts, endothelial cells, and tumor stem cells) with the help of cell type-specific promoters to assess the cell-by-cell therapeutic efficacy. The novel deregulated miRNA targets have been identified based on screening performed in patient-derived tumors that better describe the different tumor microenvironments for preclinical and clinical practices [44]. As a result, it is possible to assess tumor heterogeneity between distinct glioma cells, which demonstrate a significant degree of variability between and within tumors, as well as between individuals with glioma, and predict the likelihood of disease progression and resistance to therapy.

### 2.5. Pros and Cons of the Hydrogel

Hydrogel, as the therapeutics carrier, can enhance the drug’s penetration and retention and provide scaffolds for stem cells at the tumor site. They also help to reduce the systemic toxicity associated with the high concentrations of therapeutic agents. The pros and cons of the hydrogels are listed in Table 1. (i) The hyaluronic acid hydrogel has an easy, low cost, and rapid setup [19], and (ii) PNPPTX and MNP CpG are quickly cross-linked to form a hydrogel [22]. (iii) PCLA-PEG-PCLA can solubilize hydrophobic drugs; increase patient compliance, and decrease side effects [18,24,33]. (iv) Another pro of hydrogel nanomaterials is that they can be used for a sufficient filling of irregular damage sites, provision of a 3D cell growth environment, and improving efficacy and targetability of soluble biomolecules [18,22,26]. (v) The nature products such as gelatin, triglycerol monostearate, pig diesel, and collagen have high sensitivity, have good biocompatibility, are readily adopted by the body system, and are continuously slow released by hydrogel [25,27,29,31]. The hydrogel carriers as smart materials platforms have been developed along with the newly identified biological targets for effective glioma cancer treatments.

On the other side of the coin, the cons of the hydrogel are also shown below. The hyaluronic acid exhibits difficult real-time monitoring and genetic manipulations [19]. PLGA-PEG-PLGA shows undesirable monocyte migration, localization to systemic tissue, the difficult synchronized release of components, and a lack of safety data [18,24]. The hydrogel made by gelatin increases intracranial pressure and produces toxic degradation byproducts [25]. The cancer cell-sticky hydrogel (CSH) made by tris (2-carboxyethyl) phosphine and PEG shows low therapeutic efficiency and inevitable drug resistance [26].

The heterogeneous compositional structures of the biological tissues lead to distinctive chemical and physical processes compared to the homogeneous man-made materials, which restricts the sensitivity, accuracy, and efficiency of signal transductions through biosensor interfaces [45,46]. The mismatch in mechanical properties, including toughness, flexibility, and adhesion ability, will lead to immune rejection and disable the long-term usage of sensing devices [47,48]. in addition, whether the sensor possesses the self-healing ability will strongly affect the service life of biosensors [47]. The conductivity mismatch will lead to low efficiency and inaccurate signal collection and delivery [49]. Especially, biosystems transmit physiochemical signals through water-compliant carriers such as ions and biomolecules.

In contrast, electronic sensors rely on the controlled transportation of delocalized electrons/holes. Such mismatches at the biosensor interfaces continuously challenge the functionality of bioelectronic sensors [50]. The mismatch between the chemical diffusivity, biological tissues, and man-made materials, especially at the biosensor interface, results in signal delay and signal decay, thereby compromising the biosensor’s accuracy and functionality [51].

**Figure 3:**
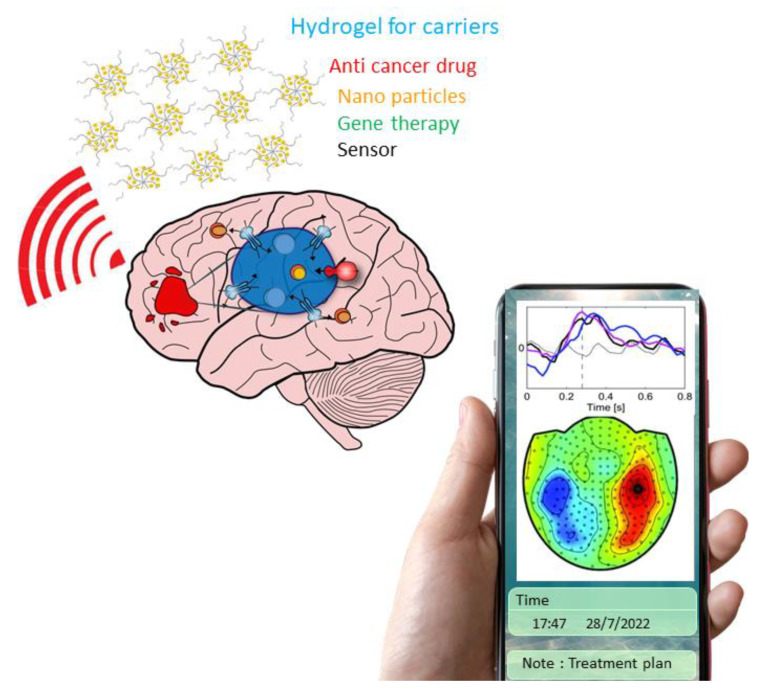
Scheme of the hydrogel as a biosensor of tissue engineering.

Current developments mainly focus on improving single or a few particularly targeted biomimetic material properties, such as self-healing and strain stiffening, corresponding to the specific purpose and the application of the sensor [52]. However, these designs often attempt to mitigate the mismatches on a case-to-case basis, while the improvement in one property often comes at the cost of another [53]. In order to achieve efficient fabrication, new research brought light to achieve efficient fabrication. Liu and his colleagues developed a silver-nanowire/PVA hydrogel/melamine sponge semidry EEG electrode for long-lasting monitoring of EEG signals [54]. Figure 3 illustrates the hydrogel sponge with nanowire printed on the wafer. The device with more peptides can be injected into the local glioma via post-surgical applications (Figure 3). Benefiting from the water storage capacity of PVA hydrogel, the electrolyte solution can be continuously released to the scalp-electrode interface during use.

The other side effects of the hydrogels can be due to their toxicity profile. The toxicity of hydrogels depends on their scaffold and the functional groups. The wide applications keep pace with the era of digitalization flexible hydrogel can serve a range of biological tissues with minimized mismatches on biosensor interfaces.

### 2.6. Hydrogel for the Biosensor of Tissue Engineering

The functioning of the human body relies on the synergistic activities of the individual tissues or organs. They are multifunctional, multi-component, multiscale, and contain a wide range of heterogeneities [55,56]. These parts, with various biochemical and biophysical properties and functions, usually have unique compositional structures, mechanical properties, electrical conductivities, and chemical diffusivities [8,57,58,59]. Biosensors, which are the nodes that translate biological information into digitalized data, are based on the communications between biological tissues and man-made materials. The third generation of neural networks has been developed to monitor the complex dynamics of neurons [60]. The asynchronous event-based information-processing in the form of spikes to resemble biology can be monitored [61]. The realistic hydrogel as a carrier for biosensors should have the capacity to carry anti-cancer drugs, nanoparticles, or genes for gene therapy and provide accurate, stable, and long-term transduce of signals across the biotic and abiotic interface. The signal can be processed with imaging, signal process, statistical analysis, and risk assessment. Health professionals can monitor the treatment progress with a cell phone (Figure 3). The hydrogels could become superlative drug delivery vehicles, surpassing the disadvantages and current limitations with the use of several conventional delivery forms and providing a promising solution for sensor applications in the future.

## 3. Conclusions

Many studies have been performed to develop new therapies that regulate tumor progression and to find new ways to treat patients with glioma precisely because of drug resistance and tumor recurrence. The review of the articles summarized that the sizes of most of the hydrogels reported are between 28 nm and 500 nm. Among the 20 articles, 80% are on using hydrogel as a post-surgical application and 65% of them employ 3D culture. The pros of the hydrogel are: quick formulation for a sufficient filling of irregular damage sites, solubilizing hydrophobic drugs, continuously slowing drug release, provision of a 3D cell growth environment, improved efficacy, targetability of soluble biomolecules, increased patient compliance, and decreased side effects. The cons of the hydrogel are: difficult real-time monitoring, genetic manipulations, the difficult synchronized release of components, and lack of safety data. The hydrogel carriers as smart materials platforms have been developed along with the newly identified biological targets for effective glioma cancer treatments. The multimodal hydrogel as the carrier can carry anticancer agents, nanomaterials, and sensor devices to treat and monitor gliomas. The flexible hydrogel should be developed to serve a range of biological tissues with minimized mismatches on biosensor interfaces. The electronic sensors can be carried out to enhance guidance of precise targeting patterns using patient-specific pathological idiosyncrasies in the future.

## 4. Methods

### 4.1. PRISMA Statement (Preferred Reporting Items for Systematic Reviews and Meta-Analyses)

**Figure 4:**
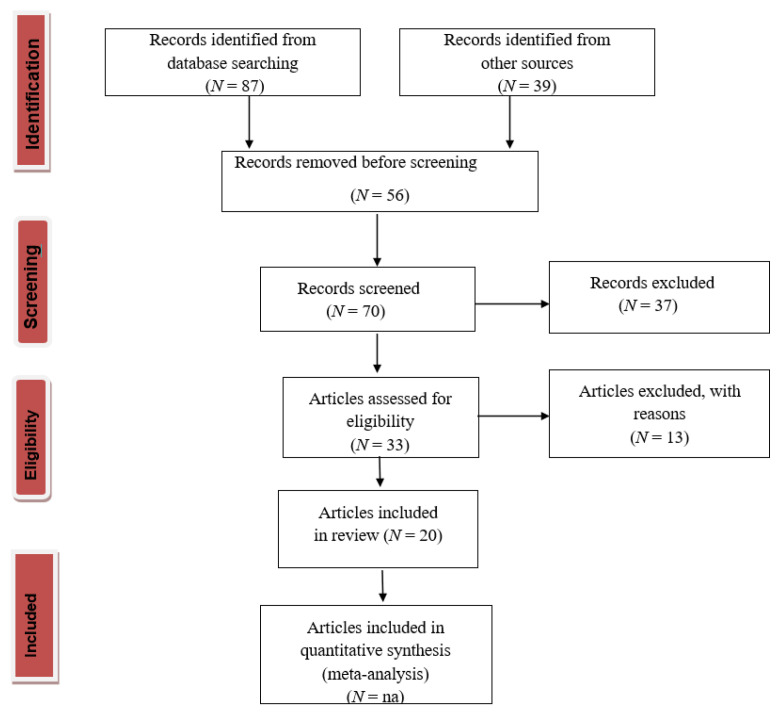
PRISMA flow diagram for literature search; na = not applicable.

We finished the PRISMA 2020 checklist and constructed a flowchart following the PRISMA guidelines and registration information. The selection process was based on the PRISMA statement 2020 [62], and the flowchart is shown in Figure 4.

### 4.2. Research Process

Hydrogel as the therapeutics carrier can enhance the drug’s penetration and retention at the tumor site. The systematic review was gathered through a literature search from online databases. Relevant articles were searched on Google Scholar and the Scopus database to identify hydrogels as carriers and pathways of drug delivery in glioma cancer. Boolean operators “AND” and “OR” were used to broaden the search. The keywords used for searching were “hydrogel” and “glioma cancer”. The article was identified through the Scopus database and Google Scholar online. The citations were collected from recent studies (2020–2022). To further ensure that we had assembled a comprehensive list of studies, we asked researchers with relevant knowledge on the topic to review and suggest keywords.

The search focused on scientific research articles using the following protocol:

i. Publication years were between 2020 and 2022.
ii. The keywords ‘‘hydrogel” AND ‘‘glioma cancer” had to appear in the title and abstract.
iii. They had to be scientific indexed papers only.

The results were screened against inclusion criteria, i.e., articles that are not relevant to the studies. The full text of papers for all the articles that fit into the inclusion criteria was retrieved.

### 4.3. Screening

Strict criteria were used to determine the relevant articles for inclusion. For example, articles were excluded if published in languages other than English or for which only an abstract was available, and then each remaining search result was grouped as one of the articles.

i. “Primary articles” research papers appeared in the peer-reviewed literature and reported original data or results based on observations and experiments.
ii. “Review” papers summarized the understanding of hydrogels as carriers and pathways of drug delivery in glioma cancer.

Throughout the screening process, the number of publications excluded in each stage and their reasons for exclusion were noted based on the guidelines outlined in the PRISMA statement 2020 in Figure 4.

## Figures and Tables

**Table 1:** A summary of hydrogel as a drug carrier for glioma cancer treatment.

Hydrogel Type	Post-Surgical	Cell Culture	Glioma Stem Cell	Drug Delivered	Particle Size (nm)	Sensor	Pros	Cons	Reference
PCL-PEG	Yes	3D	Yes	Carmustine,	597	No	Easy synthesis, safe effective treatment	N/A	[15]
Hyaluronic acid/sodium alginate	Yes	3D	Yes	Histamine	N/A	No	Sufficient filling of irregular damage sites, provision of 3D cell growth environment	N/A	[16]
PTX PLGA-NPs-loaded	Yes	3D	Yes	Paclitaxel	N/A	No	N/A	N/A	[17]
PLGA-PEG-PLGA	Yes	3D	Yes	Temozolomide	35	No	Enhance cellular internalization in GBM cells, improve low biological stability and drug’s efficacy	Undesirable monocyte migration, localization to systemic tissue	[18]
Hyaluronic acid	Yes	3D	Yes	No	N/A	No	Ease, low cost, and rapid setup	Difficult real time monitor and genetic manipulations	[19]
PVA	Yes	3D	Yes	Gemcitabine	N/A	No	Controlled and constant drug release, enhanced dosage at the targeted site	N/A	[20]
CP and CL@ RNP PTXb	Yes	3D	No	Luminol	80	No	N/A	N/A	[21]
PNPPTX and MNP CpG	Yes	3D	No	Paclitaxel	127	No	Quickly cross-linked to form a hydrogel, cellular targeted nanoparticles reach the lesion directly	N/A	[22]
Hyaluronic acid/Cucurbit	Yes	3D	No	Doxorubicin	100	No	A higher survival rate, improved drug bioavailability	N/A	[23]
PCLA-PEG-PCLA	Yes	3D	No	Paclitaxel	500	No	Solubilization of hydrophobic drugs, implanted after surgical resection of a tumor	Difficult synchronized release of components, lack safety data	[24]
Gelatin	Yes	2D	Yes	Thymidine kinase	283	No	High glioma stem cell loading capacity, more shielded from hostile resection	Increased intracranial pressure, toxic degradation byproducts	[25]
CSH: TCEP-immobilized agarose	Yes	2D	No	TCEP	N/A	No	Improved efficacy and targetability of soluble biomolecules	low therapeutic efficiency, inevitable drug resistance	[26]
Pig diemel, N,N-cyclohexyl carbide diimine, N-hydroxybutylphthalimine	Yes	2D	No	Carmustine	275	No	Readily adopted by body system, slow release, continuously released by hydrogel	N/A	[27]
Fmoc-F–FF-DOPA	Yes	2D	No	CXCL10	164	No	Low IDO1 expression, high IDO1 protein levels in tumor tissues	N/A	[28]
Triglycerol monostearate/PPS60	Yes	2D	No	Temozolomide	N/A	No	Good biocompatibility, broad cancer treatment application	N/A	[29]
Chitosan-b-glycerophosphate	Yes	2D	No	Paclitaxel	100	No	Protects niosomes against external tonicity fluctuation, prevents uncontrollable release of paclitaxel	N/A	[30]
Collagen	No	3D	No	No	N/A	Yes *	High sensitivity, nondestructive, real-time monitor of reactive oxygen species from microglial cells	N/A	[31]
PEGDA	No	3D	No	Doxorubicin	28	No	Raise local dose drug at tumor site, will not pass blood–brain barrier	N/A	[32]
PCLA-PEG-PCLA	No	3D	No	Curcumin	189	No	Increased patient compliance, decreased side effects	N/A	[33]
Hyaluronic acid	No	2D	No	Irinotecan	107	No	N/A	N/A	[34]

* Enzymatic 3D Lactate.
